# Strain, loss of time, or even gain? A systematic review of technology-based work extending and its ambiguous impact on wellbeing, considering its frequency and duration

**DOI:** 10.3389/fpsyg.2023.1175641

**Published:** 2023-07-05

**Authors:** Julia Schoellbauer, Martina Hartner-Tiefenthaler, Clare Kelliher

**Affiliations:** ^1^Department of Occupational, Economic, and Social Psychology, Faculty of Psychology, University of Vienna, Vienna, Austria; ^2^Department of Labor Science and Organization, Institute of Management Science, TU Wien, Vienna, Austria; ^3^Cranfield School of Management, Cranfield University, Cranfield, United Kingdom

**Keywords:** boundary theory, constant availability, technology-assisted supplemental work (TASW), work interruption nonwork behaviours, work-life conflict

## Abstract

Especially in knowledge-intensive professions, workers engage in work-related communication and access digital work content outside of working hours. Scientific research on technology-based work extending has flourished in recent decades, but yielded inconclusive results about its relationship with workers’ wellbeing and focused on different temporal characteristics of the behaviour. Consequently, in this article, we address the question of whether different temporal characteristics of technology-based work extending, such as the frequency and duration of the behaviour, may have different consequences for workers’ wellbeing. In the course of a systematic literature review, we analyzed 78 empirical studies published between 2007 and 2021 that investigate the relationship between the self-rated frequency and the self-rated duration of work extending behaviours and 14 wellbeing indicators. Whereas most studies examined the frequency of work extending behaviours and its consequences, only 19 studies examined the effects of its duration. Based on our findings, we propose three effects: The strain effect of frequent work extending, the gain effect of sustained work extending, and the loss-of-private-time effect inherent to work extending and independent from its frequency and duration. Our findings not only provide in-depth information on a widespread contemporary behaviour and its psychological implications, we also reveal research gaps and shed light on behaviours associated with role transitions and thus contribute to boundary theory.

## 1. Introduction

Technology-based work extending is a contemporary behavioural phenomenon with steadily increasing importance. Due to the technology-driven process of work flexibilization and the spread of mobile devices, especially knowledge workers are able to work anywhere and anytime (Messenger and Gschwind, 2016). In 2015, 23% of European workers responded to work demands in their non-work time at least several times per month (European Foundation for the Improvement of Living and Working Conditions, 2017). Three years later, a German survey revealed that 63% of the employees performed at least some regular work tasks outside their working hours (Institut zur Zukunft der Arbeit (IZA), & XING, 2018). As the COVID-19 pandemic has accelerated the prevalence of remote working from home (Eurofound, 2021), we expect that the number of workers that stay in contact with their work beyond their working hours to have grown further in the last 2 years.

Research on technology-based work extending has flourished since the introduction of the iPhone in 2007 (cnet.com, 2017). However, research so far has yielded inconclusive results on its relationship with workers’ wellbeing. For example, Park et al. (2020) and Wöhrmann and Ebner (2021) linked work extending behaviours to higher exhaustion while other studies did not find such a connection (e.g., Day et al., 2012; Piszczek, 2017). Likewise, empirical observations of an association between work extending and sleep quality yielded inconsistent results (e.g., Lanaj et al., 2016; Bowen et al., 2018). Some studies have even found evidence that work extending behaviours can be enriching for employees’ lives (Senarathne Tennakoon, 2011; Kim and Hollensbe, 2017), but others did not establish this relationship (Pangert and Schüpbach, 2014; Wan et al., 2019).

Moreover, it is striking that previous questionnaire studies operationalized technology-based work extending either in terms of its frequency (i.e., the quantity of work contacts outside working hours; e.g., Glavin and Schieman, 2010; Schieman and Young, 2010; Day et al., 2012; Park et al., 2020) or in terms of its duration (i.e., the total minutes or hours engaged in work outside working hours; e.g., Lanaj et al., 2014; Ward and Steptoe-Warren, 2014; Braukmann et al., 2018). Research indicates that the frequency and the duration of a critical or unhealthy behaviour can have different consequences for health and wellbeing (Mikulic, 2016; Yang et al., 2021; Mograss et al., 2022). Accordingly, we argue that it also seems plausible that work extending as the sum of many single work contacts outside working hours might be associated with different feelings and impressions than work extending as a certain amount (minutes, hours) of private time spent on work.

In this article, we address the research question of whether different temporal characteristics of technology-based work extending, such as the frequency and duration of the behaviour, have different consequences for various aspects of workers’ wellbeing. We aim to answer this question by conducting a systematic literature review. After systematically searching for peer-reviewed empirical studies, we compare the associations between work extending and wellbeing indicators distinguishing between studies measuring the frequency of technology-based work extending versus studies capturing its duration. Thereby, our study contributes to the literature in three ways.

First, by systematically analyzing the relationship between work extending frequency vs. its duration and wellbeing indicators, we fill a research gap and provide in-depth information on this important contemporary behaviour and its psychological implications. While work extending was operationalized either in terms of its frequency or its duration, have studies so far neglected that it could be exactly these temporal characteristics that influence the experience and thus the wellbeing of workers. However, our insights on potentially different psychological implications of work extending dependent on its high/low frequency and duration have practical implications for workers and HR managers. Moreover, our results can inform the development of intervention programs promoting workers’ wellbeing.

Second, we also shed light on behaviours associated with role transitions and thus contribute to boundary theory. Although boundary theory (Ashforth et al., 2000) describes the process of role transition as well as its antecedents and consequences, the behaviours following a role transition and their temporal characteristics have been omitted so far. Consequently, based on our findings on the potentially different consequences associated with the frequency vs. duration of technology-based work extending, we conclude that temporal characteristics need to be considered to better understand workers’ boundary management strategies. Third, by taking stock of extant studies in our systematic review, we reveal research gaps in existing literature and propose an agenda for future research.

## 2. Theoretical background

### 2.1. Work extending behaviours

Boundary theory considers work and personal life as two major life domains individuals alternate between (Ashforth et al., 2000). Within these two life domains, individuals take on multiple roles. Roles associated with work can be, for example, the role as a supervisor, a subordinate, a friend, or a co-worker, whereas the role as a spouse/partner, a parent, and a child are examples of typical roles in private life. Individuals may vary in the degree to which they segment or integrate their work and private life roles. “Segmenters” prefer clear boundaries around work and nonwork domains, while “integrators” prefer flexible and permeable boundaries. If an “integrator” engages in the role as a worker outside working hours at private places, this process of change is called role transition (Ashforth et al., 2000) or boundary crossing (Clark, 2000).

In this paper, we focus on individuals’ behaviour following their role transitions from their private role to their work role. And as engaging in a work role outside of working hours *de facto* extends the work domain at the expense of private time, we refer to this behaviour as work extending and emphasize that it is mainly enabled by the use of technological devices for work. The literature seems broadly in consensus that technology-based work extending manifests itself in activities associated with the job or the work role, for example the performance of work tasks or the professional communication with co-workers, outside of regular working hours (e.g., on workdays before or after working hours or on non-working days such as weekends or vacations) (Schieman and Young, 2010; Richardson and Thompson, 2012; Wilson, 2013; Adkins and Premeaux, 2014; Dettmers et al., 2016; Park et al., 2020).

Although it is likely that work extending behaviours and telework are carried out at the same place (i.e., at home), they differ conceptionally. Work extending activities take place outside working hours and thus during a time when the individual is normally engaged in a private role. Moreover, as work extending is not contracted and thus often not paid (Duxbury and Smart, 2011), it should be distinguished from on-call work and long working hours.

### 2.2. Work extending behaviours and wellbeing

Drawing on the fourth proposition of boundary theory (Ashforth et al., 2000), “the greater the role integration, the greater the potential for confusion regarding which role identity to enact and for undesired interruptions” (p. 481), most studies so far hypothesized detrimental consequences for workers’ wellbeing following work extending (see also Schöllbauer et al., 2021). However, empirical research so far has yielded inconclusive findings regarding its implications for burnout (e.g., Piszczek, 2017; Park et al., 2020), sleep quality (e.g., Lanaj et al., 2016; Bowen et al., 2018), and work-to-nonwork enrichment (e.g., Pangert and Schüpbach, 2014; Kim and Hollensbe, 2017), indicating that boundary theory’s proposition may not be sufficient to predict the consequences of this contemporary phenomenon. Schlachter et al. (2018) concluded that work extending is “not inherently ‘good’ or ‘bad’, but a complex matter.” (p. 840). With this article, we aim to reduce the complexity of work extending’s psychological implications by grasping it as behavioural phenomenon with different temporal characteristics (Roe, 2008; Fisher et al., 2021). Generally, behaviours can be described in terms of their frequency (e.g., How often do you engage in a certain behaviour?) and duration (e.g., How much time do you invest in a certain behaviour?). Various behaviours have been empirically observed in terms of their frequency and duration, for example napping behaviour (Mograss et al., 2022), face-touching behaviour (that fosters infection; Keller et al., 2021), and smartphone usage behaviour (Wilcockson et al., 2018; Shaw et al., 2022). Although there is a small number of studies that captured both temporal characteristics (i.e., Richardson and Thompson, 2012; Ward and Steptoe-Warren, 2014; Minnen et al., 2021), most studies on work extending have operationalized technology-based work extending either in terms of its frequency or of its duration.

Research indicates that the frequency of a critical or unhealthy behaviour has different psychological implications than its duration. Yang et al. (2021) associated only the frequency of smartphone use with a smartphone addiction and Mikulic (2016) reported that the higher the frequency of smartphone use, the higher the strain experienced and the lower the level of happiness experienced by the user. Moreover, in sleep research, it was shown that only the frequency of napping throughout the day had a significant impact on sleep quality on the same day, but not the duration of napping on a day (Mograss et al., 2022).

Although boundary theory (Ashforth et al., 2000) describes the process of role transition as well as its antecedents and consequences for the primary life role, the behaviours following a role transition and their temporal characteristics remain unexplored so far. As the temporal characteristics of a behaviour can have different psychological consequences (i.e., cognitions, emotions, and subsequent behaviours), we argue that technology-based work extending has different consequences for workers’ wellbeing dependent on its temporal characteristic (i.e., its frequency and its duration). Thereby, for example, only a higher frequency of work extending might be associated with serial interruptions during private hours, and interruptions are known to relate to negative affect such as feeling distressed, upset, and irritable (Sonnentag, 2018). Moreover, as the duration indicates the amount of time work “steals away” from private life, one could argue that especially a higher duration of work extending behaviours might be associated with a temporal conflict between work and private life (Greenhaus and Beutell, 1985). Consequently, we state the following research question:

*RQ*: Do the frequency and the duration of technology-based work extending have different implications for workers’ wellbeing?

To answer the research question, we systematically searched for articles reporting studies that correlated work extending’s frequency or duration and compared with various indicators for workers’ wellbeing.

## 3. Method

Since there is already a certain amount of empirical work observing the association between technology-based work extending and wellbeing (Ďuranová and Ohly, 2016; Schlachter et al., 2018; Schöllbauer et al., 2021), we chose the systematic review method to answer our research question. Thereby, we followed a scientific, replicable and transparent selection process (Centre for Reviews and Dissemination, University of York, 2009) and summarized as well as synthesized research evidence on the given topic area (Daniels, 2019). We collected peer-reviewed empirical evidence from questionnaire studies investigating technology-based work extending and its relationship with workers’ wellbeing between 2007 and 2021. We chose this period to cover the effect of the emergence and widespread distribution of smartphones in first world countries, starting with the launch of the iPhone in 2007 (cnet.com, 2017). Moreover, we only focused on questionnaire studies in order to be able to systematically cluster different temporal characteristics of work extending and wellbeing indicators.

Two reasons informed our decision to conduct a systematic literature review, rather than a meta-analysis: First, the measurements applied for technology-based work extending are characterized by heterogeneity (see also Schlachter et al., 2018). “Heterogeneity is a critical issue in meta-analysis because it implies the appropriateness of combining the collected studies and impacts the reliability of the synthesized results” (Lin, 2020, p. 376). Second, with regard to our focus on work extending’s temporal characteristics and their relationship with certain wellbeing indicators, we had to calculate with small sub-samples (partly only from one study) which contradicts the idea of a meta-analysis. Nevertheless, we systematically collected, analyzed, and present quantitative findings to identify research gaps and potential research avenues.

To address our research question (Do studies focusing on the frequency of work extending activities yield other association with indicators for workers’ wellbeing as compared to studies focusing on the duration of work extending behaviours?) we counted and compared the relative amount of negative, positive, and not significant correlations between work extending’s frequency and duration with wellbeing indicators.

### 3.1. Literature selection

**Figure 1:**
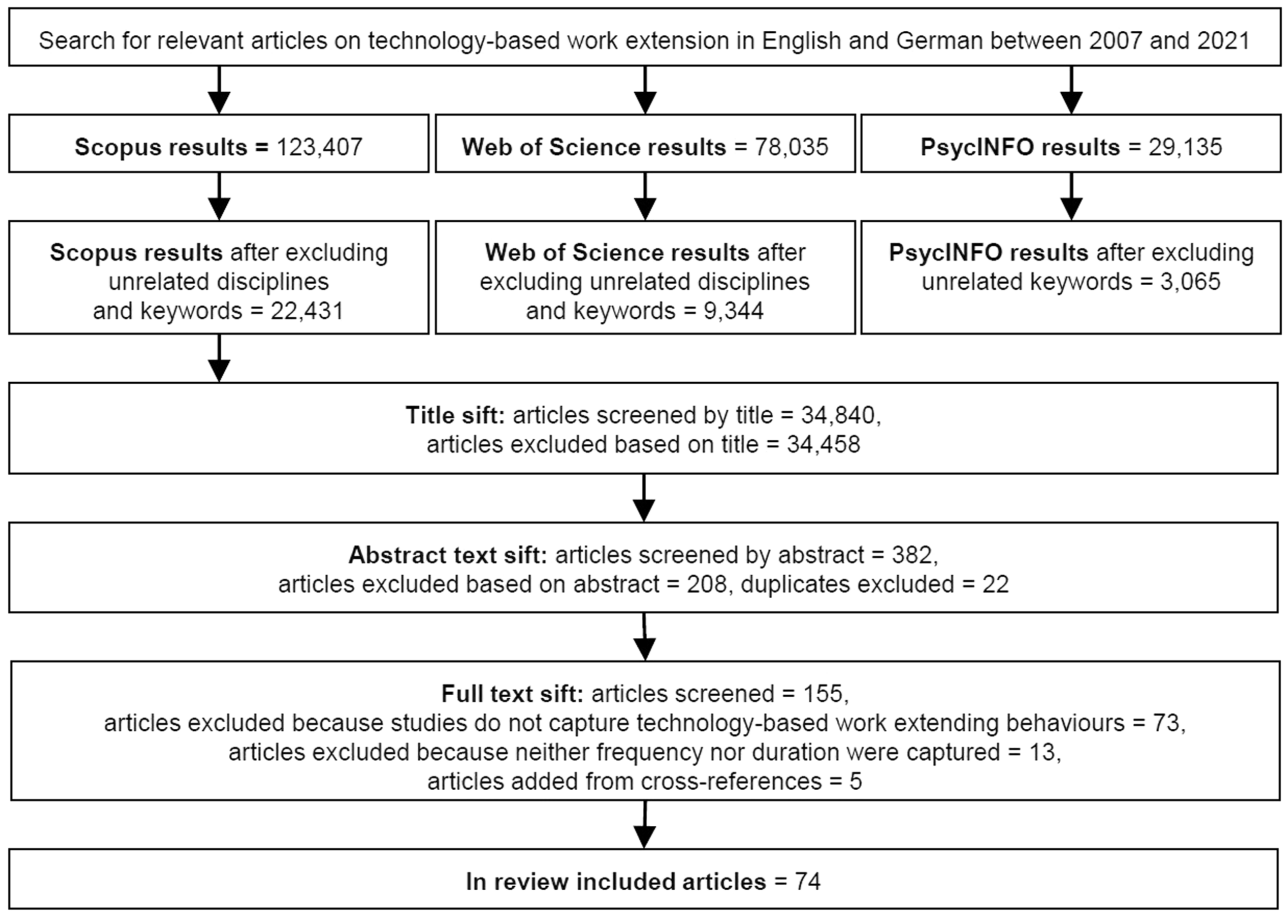
Systematic review flow chart.

Selecting the literature to review, we followed the PRISMA (i.e., preferred reporting items for systematic reviews and meta-analyzes) guidelines for systematic reviews (Page et al., 2021). First of all, we identified relevant keywords in English and German describing our research focus clustered into four main areas of interest: (1) working individuals, (2) technology-based, (3) extended contact to work, and (4) investigated by means of quantitative studies. A list of keywords used for the literature search is provided in Table 1. Our search covered title, abstract and keywords using the multidisciplinary online databases Scopus, Web of Science, and PsycINFO. As illustrated in the PRISMA flow diagram illustrating the search process in Figure 1, we initially found a total of 230,577 pieces of literature. After excluding unrelated disciplines (e.g., medicine, environmental science, informatics) and keywords (e.g., disease, sustainability, robotics), we sifted a total of 34,840 peer-reviewed pieces resulting in 74 pieces of literature for our review, including 69 journal articles (2 in German, 67 in English) and five dissertations (Fender, 2010; Senarathne Tennakoon, 2011; Wilson, 2013; Moore, 2017; Schlachter, 2017), covering multiple scientific disciplines such as psychology, sociology, and management. For simplicity reasons, we will hereinafter refer to all pieces of literature reviewed as “articles.”

**Table 1:** Keywords used for systematic literature search.

Area of interest	Keywords
1. Relation to work	Employee* OR Manager* OR “Professionals” OR Worker* OR “Working-individual” OR Arbeitnehmer* OR Angestellte* OR Berufstätige* OR Beschäftigte* OR Erwerbstätige*
AND 2. Technology-enabled connection with work	Accessib* OR “Additional-work” OR Availab* OR Call* OR Cellphone* OR “Cell-phone” OR Communic* OR Computer* OR Connect* OR Contact* OR Email* OR “E-Mail” OR Messag* OR Messenger* OR Mobilephon* OR “Mobile-phone” OR Notebook* OR Phone* OR Reachab* OR Respon* OR Telephone* OR Smartphon* OR “Supplemental-work” OR Technolog* OR Anruf* OR Erreichbar* OR Handy* OR Kommuni* OR Kontakt* OR Mobiltele* OR Nachrichten OR Verfügbar*
AND 3. Time extension of work	“After-hour” OR “After-normal” OR “After-regular” OR “After-work” OR “Beyond-hours” OR “Beyond-normal” OR “Beyond-regular” OR “Beyond-work” OR Boundaryless* OR “Boundary-spanning” OR Blurr* OR Constant* OR Continu* OR “Day-off” OR “Days-off” OR Evening* OR Expan* OR Exten* OR Family* OR “Free-time” OR Holiday OR home* OR Integrating OR Leisure* OR “Life-domain” OR Night* OR Non-work* OR “Off-work” OR Ongoing* OR “Outside-of” OR “Private-domain” OR “Private-hours” OR “Private-life” OR “Private-time” OR Tether* OR “Time-off” OR Vacation* OR Weekend* OR Abend* OR “Ausserhalb-der-Arbeit” OR Durchgehend* OR Durchlässig* OR Erweiter* OR Familie OR Feierabend* OR Freizeit* OR Grenzenlos* OR Konstant* OR “Nach-der-Arbeit” OR Nacht* OR Privatleben OR Privatzeit* OR Ständig* OR Urlaub* OR Wochenende*
AND 4. Empirical studies	Questionnaire* OR Study* OR Studies OR Survey* OR Fragebogen* OR Studie*
AND NOT 5. Unrelated keywords	Adolescent* OR Adulthood* OR Aged* OR Aging* OR Animal* OR Apartheid* OR Artificial* OR Asyl* OR Athlet* OR Autism* OR Blockchain* OR Brand* OR Bullying* OR Cancer* OR Cerebral* OR Childcare OR “Child-care” OR Childhood* OR “Child-welfare” OR Clinic* OR Consum* OR Crimin* OR Crowd* OR Dement* OR Diabetes* OR Discrimination* OR Disease* OR Disability* OR Disorder* OR Divorce* OR Drug* OR “E-commerce” OR “E-Learning” OR Elder* OR Entrepreneur* OR Farm* OR Father* OR Football* OR “Foster-Care” OR Funeral* OR Game* OR Gamification* OR Grandchild* OR Hack* OR Healthcare* OR “Health-care” OR Hospital* OR Immigra* OR Infant* OR Injury* OR Islam* OR Juvenile* OR “Machine-Learning” OR Migra* OR Mother* OR Music* OR Neural* OR Nurs* OR Nutrition* OR “Older-adults” OR Outdoor* OR Palliativ* OR Parent* OR Patent* OR Patient* OR Pedagog* OR Perinatal* OR Postnatal* OR Postpartum* OR Posttrauma* OR Pregnan* OR Presenteism* OR Refugee* OR Religi* OR Reproduct* OR Robotic* OR School* OR Security* OR Sex* OR “Social-work” OR “Social-worker” OR Stepparent* OR Student* OR Sustainab* OR Talent* OR Teachers OR Touris* OR Trauma* OR Traveler* OR Truck* OR Undergraduate* OR Vaccin* OR Victim* OR Violen* OR Voter* OR X-Ray*

The search string in each area of interest consists of English and German keywords, consecutively. All four areas of interest were linked with the command “and” in the search engines. An asterisk indicates that keywords with different endings are included in the search, a quotation mark marks a bound search string which is not to be altered by the search engine.

We excluded 13 papers because they did not measure the behaviour’s frequency or duration but simply whether or not the worker exhibits the behaviour (Park and Jex, 2011; Ohly and Latour, 2014; Ragsdale and Hoover, 2016; Manapragada, 2017; Wang et al., 2017; Poethke et al., 2019; Büchler et al., 2020) or by measuring some kind of general character of the behaviour with Kossek et al.’s (2012) work interrupting nonwork behaviours scale (Kossek et al., 2012; Wright et al., 2015; Kinnunen et al., 2016, 2017; Palm et al., 2016; Russo et al., 2018). Finally, we added five pieces of literature (Richardson and Thompson, 2012; Chen and Karahanna, 2014; Ward and Steptoe-Warren, 2014; Schlachter, 2017; Santarpia et al., 2021) that were not indexed in the search engines used, but were cited in other articles we reviewed.

The findings from the 74 articles considered were based on a total of 78 questionnaire-based studies which captured technology-based work extending behaviours and at least one wellbeing indicator of in total 124,470 workers. The lowest sample size (i.e., 39 workers recruited via Facebook and LinkedIn) was reported by Yeh et al. (2020) who conducted a daily diary study over the course of 10 days. The highest sample size was reported by Arlinghaus and Nachreiner (2014) who analyzed the data of the fourth and fifth European Working Conditions Survey (EWCS 2005 and 2010), including 22.836 and 34.399 employed workers. Among the 78 studies, technology-based work extending was represented 81 times as a variable measured through 40 distinct measurements. Only two studies reported an alpha reliability of below 0.70 (i.e., two items yielded an alpha of 0.60; Albertsen et al., 2010; three items yielded an alpha of 0.65; Glavin and Schieman, 2010). The four items applied in the study of Piszczek (2017) yielded the highest alpha coefficient of 0.95. The studies empirically examined a total of 181 relationships between technology-based work extending frequency or duration and a total of 14 wellbeing indicators (148 between-person relations and 33 within-person relations).

### 3.2. Grouping work extending’s temporal characteristics

**Figure 2:**
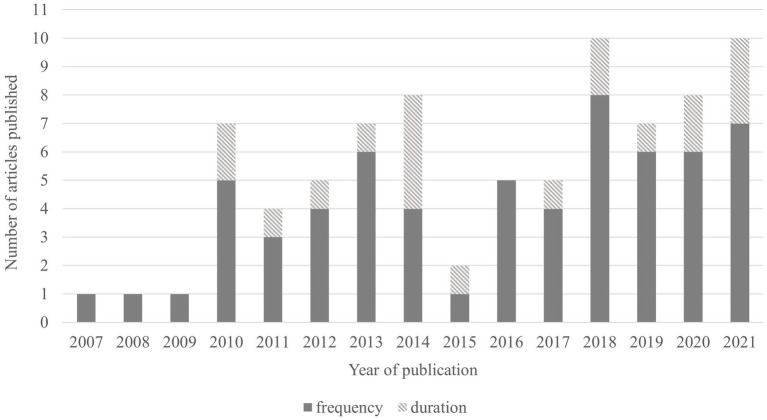
Research interest between 2007 and 2021 (sum of articles reviewed per publication year, categorized by means of the temporal characteristic of technology-based work extending behaviours captured).

Depending on how work extending behaviours was measured (questionnaire items and answer scales), we clustered the findings into frequency and duration of technology-based work extending behaviours. As illustrated in Figure 2, using frequency was by far the most popular way to capture work extending. More precisely, of the 74 studies considered in this review, 62 studies measured work extending behaviours by means of their frequency. In most of these studies (i.e., 53), participants were asked directly about how often they engage in work extending behaviours. A typical questionnaire item was phrased as a question beginning with “How often did you…” and was accompanied by answer scales ranging from “never” to “several times a day” (e.g., Glavin and Schieman, 2010; Schieman and Young, 2010; Wilson, 2013) or from “never” to a more ambiguous “very often” or “always” (e.g., Day et al., 2012; Bowen et al., 2018; Xie et al., 2018). Seven additional studies were grouped by frequency because the participants either agreed or disagreed with frequency statements such as frequently (e.g., Chen and Casterella, 2019), often (Park et al., 2020), or intensively (e.g., Gombert et al., 2018a,b). Finally, McDaniel et al. (2021) and Minnen et al. (2021) captured the frequency of work extending by asking participants to indicate the total number of their work contacts in a specific time period.

In 19 studies, work extending behaviours were measured by means of their duration. In nearly all of these studies, participants were asked to estimate the total number of minutes or hours they engage in work extending on a typical workday, in a typical workweek, or on a specific day. Thus, participants answered either by means of an open response format (e.g., Senarathne Tennakoon, 2011; Lanaj et al., 2014; Braukmann et al., 2018), or on a scale ranged from “none” or “0 min” to “eight hours or more” (e.g., Adkins and Premeaux, 2014; Ward and Steptoe-Warren, 2014; Thörel et al., 2020). Only one study asked participants about the subjective extent of their work extending behaviours by asking them to rate statements (e.g., “In the last 2 weeks, I did work tasks during home time”) with the help of a Likert-scale ranging from “not at all” to “very much” (Kim and Hollensbe, 2017).

### 3.3. Grouping wellbeing indicators and dimensions

In total, 181 correlations between work extending and wellbeing were investigated. Based on semantic similarities of the items used to measure various aspects of wellbeing, we clustered the 181 wellbeing measures into 14 wellbeing indicators. For example, we grouped wellbeing measures that captured “emotional exhaustion” (e.g., Day et al., 2012; Dettmers et al., 2016), “fatigue” (e.g., Fender, 2010; Arlinghaus and Nachreiner, 2014), “ego depletion” (e.g., Lanaj et al., 2014; Gombert et al., 2018a,b), and “low activated unpleasant affect” (Schlachter, 2017) together under the wellbeing indicator exhaustion as they all measured workers’ low activated negative feelings of exhaustion and fatigue. Based on the framework of occupational wellbeing stated by Van Horn et al. (2004), we grouped the 14 wellbeing indicators by the dimensions affective, cognitive, social, and psychosomatic wellbeing. Additionally, we included recovery consisting of the recovery experiences described by Sonnentag and Fritz (2007) as well as sleep (Sonnentag, 2018) as a fifth dimension since it is an important literature stream in work and organisational psychology (see Tables 2–6 for more detail).

**Table 2:** Grouping of affective wellbeing indicators.

Wellbeing indicator	Original name of wellbeing-related study variable	References
Enthusiasm	Happiness	Butts et al. (2015)
	High-activation pleasant affect	Schlachter (2017)
	Positive affect	Khalid et al. (2021), Reinke and Ohly (2021)
	Vigor	Eichberger et al. (2021), Minnen et al. (2021)
	Work-based resource gain	Wan et al. (2019)
Exhaustion	Burnout	Ferguson et al. (2016)
	Cognitive weariness	Hu et al. (2019)
	Daily job stress	Yeh et al. (2020)
	Ego depletion	Gombert et al. (2018b), Lanaj et al. (2014)
	Emotional exhaustion	Day et al. (2012), Dettmers et al. (2016), Piszczek (2017), Tang et al. (2019), Thörel et al. (2020), Thörel et al. (2021), Xie et al. (2018), Zhang et al. (2021)
	Exhaustion	Chen and Karahanna (2018), Wepfer et al. (2018)
	Fatigue	Arlinghaus and Nachreiner (2014), Fender (2010), Minnen et al. (2021), Schlachter (2017)
	Job burnout	Leung (2011), Park et al. (2020), Wright et al. (2014)
	Low-activation unpleasant affect	Schlachter (2017)
	Need for recovery	Gombert et al. (2018b)
	Psychological distress	Glavin et al. (2011)
	Work-based resource loss	Wan et al. (2019)
Serenity	Low-activation pleasant affect	Schlachter (2017)
Strain	Affective rumination	Minnen et al. (2021), Schlachter (2017)
	Anger	Butts et al. (2015)
	High-activation unpleasant affect	Schlachter (2017)
	ICT perceived stress	Day et al. (2012)
	Interruption overload	Chen and Karahanna (2018)
	Job stress	Wilson (2013)
	Negative affect	Khalid et al. (2021), Park et al. (2020), Reinke and Ohly (2021)
	Perceived life stress	Wright et al. (2015)
	Psychological distress	Bowen et al. (2018), Schieman and Young (2013)
	Stress and irritability	Arlinghaus and Nachreiner (2014)
	Telestressor-overload	Barber and Jenkins (2013)

**Table 3:** Grouping of cognitive wellbeing indicators.

Wellbeing indicator	Original name of wellbeing-related study variable	References
Problem-solving pondering	Problem-solving pondering	Minnen et al. (2021)
Psychological enrichment	Work-to-family enrichment	Wan et al. (2019)
	Work-to-home positive spillover	Kim and Hollensbe (2017)
	Work-to-nonwork enrichment	Senarathne Tennakoon (2011)

**Table 4:** Grouping of psychosomatic wellbeing indicators.

Wellbeing indicator	Original name of wellbeing-related study variable	References
Psychosomatic health complaints	Headaches	Arlinghaus and Nachreiner (2014)
	Musculoskeletal problems	Arlinghaus and Nachreiner (2013)
	Psychosomatic health complaints	Wöhrmann and Ebner (2021)
	Somatic health complaints	Wilson (2013)
	Stomach ache	Arlinghaus and Nachreiner (2014)

**Table 5:** Grouping of recovery indicators.

Wellbeing indicator	Original name of wellbeing-related study variable	Reference
Feeling recovered	Recovery experience	Zhang et al. (2021)
Perceived control over life	Boundary control	Wilson (2013)
	Control over off-job activities	Dettmers et al. (2016)
	Techno-invasion	Leung (2011)
Psychological detachment	Boundary-spanning thoughts	Schieman and Young (2010)
	Erholungsunfähigkeit [inability to recover]	Rau and Göllner (2019)
	Negative work rumination	Park et al. (2020)
	Psychological detachment	Barber and Jenkins (2013), Braukmann et al. (2018), Dettmers et al. (2016), Eichberger et al. (2021), Hu et al. (2019), Mellner (2016), Park and Jex (2011), Reinke and Ohly (2021), Richardson and Thompson (2012), Thörel et al. (2020), Thörel et al. (2020), Thörel et al. (2021), Ward and Steptoe-Warren (2014)
	Psychological transition	Chen and Karahanna (2018)
Sleep quality	Insomnia	Park et al. (2020)
	Sleep problems	Arlinghaus and Nachreiner (2014), Bowen et al. (2018), Schieman and Young (2013), Thörel et al. (2020), Thörel et al. (2021)
	Sleep quality	Braukmann et al. (2018), Gombert et al. (2018a), Hu et al. (2019), Lanaj et al. (2014)
Sleep quantity	Sleep quantity	Barber and Jenkins (2013), Hu et al. (2019), Lanaj et al. (2014)

**Table 6:** Grouping of social wellbeing indicators.

Wellbeing indicator	Original name of wellbeing-related study variable	References
(Private) life satisfaction	Family satisfaction	Leung (2011)
	Life satisfaction	McCloskey (2016), McDaniel et al. (2021), Wilson (2013)
	Marital satisfaction	Zhang et al. (2021)
Conflict between work and private life	Work interference with family work interference with personal life	Jostell and Hemlin (2018), Richardson and Thompson (2012) Moore (2017)
	Work–family conflict	Adkins and Premeaux (2014), Bowen et al. (2018), Cho et al. (2020), Glavin and Schieman (2010), Adkins and Premeaux (2014), Hecht and Allen (2009), Khalid et al. (2021), McCloskey (2016), Schieman and Young (2010, 2013), Thörel et al. (2020), Ward and Steptoe-Warren (2014), Wilson (2013)
	Work-home spillover	Berkowsky (2013), Leung (2011)
	Work-life balance	Chen and Casterella (2019), Tang et al. (2019), Wepfer et al. (2018)
	Work-life conflict	Kotecha et al. (2014), van Zoonen et al. (2020), Wright et al. (2014)
	Work-to-family conflict	Albertsen et al. (2010), Fender (2010), Fenner and Renn (2010), Glavin and Schieman (2010), Leung (2011), Matthews et al. (2010), Nevin and Schieman (2021), Santarpia et al. (2021), Wan et al. (2019), Yang et al. (2021)
	Work-to-family spillover	McDaniel et al. (2021), Wajcman et al. (2010)
	Work-to-home conflict	Delanoeije et al. (2019), Gadeyne et al. (2018), Kim and Hollensbe (2017), Schieman and Glavin (2008)
	Work-to-life conflict	Boswell and Olson-Buchanan (2007), Diaz et al. (2012)
	Work-to-nonwork conflict	Butts et al. (2015), Chen and Karahanna (2014), Senarathne Tennakoon (2011)

### 3.4. Data analysis

To compare the studies, we evaluated between-person correlation coefficients or regression coefficients (if the correlation information was not provided) derived from cross-sectional and longitudinal studies, as well as within-person correlation coefficients or regression coefficients (if the correlation information was not provided) derived from diary studies with repeated measurements. Between-person relationships were reported by most studies and refer to workers’ experiences compared to the experiences of other workers. Within-person relationships indicate the experience of a specific worker on a particular day in relation to the same worker’s experiences averaged from all measurement occasions. More specifically, we added up the number of significantly negative, significantly positive, and nonsignificant relationships between work extending (measured in terms of its frequency vs. duration) and the 14 wellbeing indicators. Table 7 presents an overview of our analysis, revealing a pattern of relationships which we describe in the results section.4.

**Table 7:** Relationship between technology-based work extending and wellbeing in relation to the behaviours temporal characteristics (frequency vs. duration).

Wellbeing indicator	frequency			duration		
	−	NO	+	−	NO	+
Affective wellbeing						
exhaustion	0/0	8/1	14/5	0/0	8/1	1/1
strain	0/0	3/1	10/3	0/0	2/1	1/2
serenity	0/1	1/1	0/0	0/0	0/0	0/0
enthusiasm	0/1	3/3	2/0	0/1	2/2	0/0
Cognitive wellbeing						
problem-solving pondering	0/0	1/1	0/0	0/0	1/0	0/1
psychological enrichment	0/0	1/0	0/0	0/0	0/0	2/0
Psychosomatic wellbeing						
psychosomatic health complaints	0/0	1/0	6/0	0/0	0/0	0/0
Recovery						
feeling recovered	2/0	0/0	0/0	0/0	0/0	0/0
perceived control over life	3/0	0/0	0/0	0/0	0/0	0/0
psychological detachment	12/2	0/0	0/0	6/2	0/0	0/0
sleep quantity	6/1	2/1	0/0	1/0	0/3	0/0
sleep quality	2/0	0/0	0/0	0/2	0/0	0/0
Social wellbeing						
conflict between work and private life	0/0	3/0	32/2	1/0	1/0	8/1
(private) life satisfaction	1/0	3/0	2/0	0/0	0/0	0/0

The numbers indicate the sum of studies yielding significantly negative (−), positive (+) and nonsignificant (no) linear relationships between recurring or continuous work extending and the respective wellbeing indicator. On the left side of the slash is the sum of between-person findings, on the right side of the slash is the sum of within-person findings yielded by diary studies.

## 4. Results

The findings presented below are a narrative synthesis of the 78 studies reviewed, which involved a total of 124,840 working individuals. In sum, 59 studies captured only the frequency and 16 captured only the duration of participants’ work extending behaviours. Three studies captured both the frequency and the duration. It is worth mentioning that none of the studies included in this systematic review explored indicators for all five wellbeing dimensions. Table 7 summarizes the findings by showing the sum of studies yielding significantly negative and positive, as well as nonsignificant, correlations between the measurement of technology-based work extending and a wellbeing indicator. Following Daniels (2019), we present the empirical findings in the next sections for each wellbeing dimension separately by means of evidence statements.

### 4.1. Affective wellbeing

The majority of studies linking work extending and *negative affect* yielded a positive relationship with the behaviour’s frequency and no relationship with its duration. More precisely, it seems that workers who experience more frequent work extending tend to report higher levels of negative affect in terms of strain (e.g., Schieman and Young, 2013; Chen and Karahanna, 2018) and exhaustion (e.g., Wan et al., 2019; Park et al., 2020), compared to workers who experience less frequent work extending. The positive association of frequent work extending with negative affect was also shown on a daily within-person level (strain: Schlachter, 2017; Gombert et al., 2018a,b; exhaustion: Schlachter, 2017; Park et al., 2020). All studies investigating strain or exhaustion on a daily within-person level found significant positive correlations with frequency. Compared to studies finding a significant positive relationship, fewer studies found no relationship of work extending’s frequency with feelings of strain (e.g., Schlachter, 2017; Minnen et al., 2021) and exhaustion (e.g., Day et al., 2012; Piszczek, 2017), and all of these studies investigated the relationship at the between-person level. With regard to work extending’s duration, the findings were the other way around, with an equal number of studies yielded a positive relationship (Butts et al., 2015; Minnen et al., 2021) and no relationship with strain (Wright et al., 2015; Reinke and Ohly, 2021). Regarding exhaustion, the majority of studies showed no relationship (e.g., Fender, 2010; Lanaj et al., 2014), whereas fewer studies showed a positive relationship with feelings of exhaustion (Minnen et al., 2021).

With regard to workers’ *positive affect*, the majority of empirical studies yielded no significant relationship with both of the temporal characteristics of the behaviour. More precisely, frequent work extending was not associated with feelings of enthusiasm (Schlachter, 2017; Eichberger et al., 2021; Minnen et al., 2021) or of serenity (Schlachter, 2017) at the person-level or at the daily within-person level. With regard to the behaviour’s duration, a similar picture emerged as neither Butts et al. (2015), Minnen et al. (2021), or Reinke and Ohly (2021) found a significant correlation between work extending’s duration and enthusiasm. We found no study linking duration with serenity.

### 4.2. Cognitive wellbeing

We found only four studies that examined the relationship between work extending and (two indicators of) cognitive wellbeing. However, this limited evidence yielded no correlations with the behaviour’s frequency and largely positive correlations with its duration. First, Minnen et al. (2021), one of the few studies that examined both the frequency and the duration of workers’ work extending behaviours, found no association between work extending’s frequency and problem-solving pondering, neither on the between-person nor on the daily within-person level. As a form of rumination, problem-solving pondering is characterized by continued thoughts about unresolved work matters outside working hours with the aim of finding a solution to them, without negative affective activation (Cropley and Zijlstra, 2011). Minnen et al. (2021) further reported that work extending’s duration was positively linked at the daily within-person level, but not on the between-person level.

Second, Wan et al. (2019) reported that there was no link between work extending’s frequency and workers’ psychological enrichment, but Kim and Hollensbe (2017) and Senarathne Tennakoon (2011) both found that workers who reported higher duration of work extending also reported feeling more enriched by work. Psychological enrichment refers to the “positive spill-over” (Kim and Hollensbe, 2017, p. 98) of cognitive skills and positive emotions acquired at work that enrich the workers in their character and thus change their cognition, not only at work, but also in their private lives in a positive way (Greenhaus and Powell, 2006; Hanson et al., 2006).

### 4.3. Psychosomatic wellbeing

A total of four studies linked work extending behaviours to psychosomatic wellbeing indicators, and they all captured frequency. Three out of four studies linked work extending’s frequency to an increase of musculoskeletal problems (Arlinghaus and Nachreiner, 2013), headache, stomach ache (Arlinghaus and Nachreiner, 2014), and to more general psychosomatic health complaints (Wöhrmann and Ebner, 2021). Wilson (2013) found no association to work extending’s frequency by more generally asking about participants’ somatic health complaints. No study to date linked the duration of work extending behaviours to psychosomatic wellbeing indicators.

### 4.4. Recovery

The majority of studies examining the link between work extending and indicators of a successful mental recovery from work outside working hours yielded a detrimental relationship with both temporal characteristics. More precisely, all studies investigating the relationship between frequency and workers’ feeling of having recovered (Zhang et al., 2021), perceived control over life (e.g., Wilson, 2013; Dettmers et al., 2016), psychological detachment from work outside working hours (e.g., Ward and Steptoe-Warren, 2014; Mellner, 2016), and sleep quantity (Barber and Jenkins, 2013; Hu et al., 2019) reported negative correlations. Moreover, a total of five studies linked frequency to lower levels of workers’ sleep quality (e.g., Bowen et al., 2018; Hu et al., 2019), but two found no association (Gombert et al., 2018a; Thörel et al., 2020). Psychological detachment (e.g., Rau and Göllner, 2019; Thörel et al., 2020) and sleep quantity (Lanaj et al., 2014) were also detrimentally linked to work extending’s duration. Psychological detachment was linked to duration on the between-person, as well as on the daily within-person level. The sleep quantity-duration association was only investigated on the daily level. Regarding sleep quality, diary studies yielded no correlation with work extending’s duration on the daily level (Lanaj et al., 2014; Braukmann et al., 2018), whereas a longitudinal study showed that workers who experienced a higher frequency of work extending reported lower sleep quality, compared to workers who reported less frequent work extending (Thörel et al., 2021). No study linked the duration of work extending behaviours to the recovery indicators of perceived control over life and feeling recovered.

### 4.5. Social wellbeing

The majority of studies linking work extending and social wellbeing indicators yielded a detrimental relationship with both temporal characteristics. Findings from our review indicate that workers who report engaging in work extending behaviours at a certain frequency (e.g., Fenner and Renn, 2010; Kotecha et al., 2014) and duration (e.g., Adkins and Premeaux, 2014; Kim and Hollensbe, 2017) also perceive a greater conflict between work and private life, both on the between-person and on the within-person level. Regarding workers’ (private) life satisfaction, the evidence is less clear: McDaniel et al. (2021) reported a negative relationship, whereas others showed no relationship (Wilson, 2013; McCloskey, 2016) for women (Zhang et al., 2021), and finally Leung (2011) and Zhang et al. (2021) reported a positive association with workers’ (private) life satisfaction (the latter only for men). We did not find any study linking work extending’s duration to workers’ satisfaction with their private life or life in general.

## 5. Discussion

In line with prior research, we regard technology-based work extending not as a rigid job characteristic, but as contemporary behavioural phenomenon that has various dynamic features (Roe, 2008; Fisher et al., 2021). More precisely, we analyzed whether different temporal characteristics of technology-based work extending behaviours show different relationships with indicators of workers’ wellbeing extending prior reviews of this phenomenon (Ďuranová and Ohly, 2016; Schlachter et al., 2018; Schöllbauer et al., 2021). Based on this analysis, we propose three effects linking work extending behaviours and workers’ wellbeing: the *strain effect* triggered by frequent work extending behaviours and indicated by higher levels of strain and exhaustion, as well as lower sleep quality; the *gain effect* triggered by sustained work extending behaviours and indicated by cognitive skills acquisition and problem-solving pondering; and the *loss-of-private-time effect* triggered by frequent and sustained work extending behaviours and indicated by a conflict between work and private life, lower sleep quantity, and less time thinking about work (a.k.a. low levels of psychological detachment).

Building on boundary theory (Ashforth et al., 2000), we take a time perspective and expend it by shedding light on the role of frequency and length of role transition. Boundary theory (Ashforth et al., 2000) focuses on individuals’ micro role transitions (e.g., from being a mother/father at dinner with the family to being a worker who takes a work-related call). Although boundary theory argues the importance of role context, it neglects temporal aspects. Depending on the time span the worker remains in the new role, we assume different interferences from the previous role. In line with this idea, our findings reveal that temporal patterns of role transitions potentially shape distinctive effects on wellbeing, which are explained below.

### 5.1. The strain effect of frequent work extending

Studies that operationalized work extending using frequency measures showed relationships with higher strain and exhaustion and lower sleep quality. For work extending’s duration, however, mostly nonsignificant correlations with strain, exhaustion, and sleep quality were yielded. Boundary theory (Ashforth et al., 2000) emphasizes the interruptive character of role transitions and frequent work extending behaviours may be unwanted interruptions of the private life role being experienced. “Interruptions, as role boundary violations, disrupt the enactment of a role identity and may force an unwanted shift to another role identity” (Ashforth et al., 2000, p. 481). Accordingly, working outside of working hours has been described as work-related interruptions during personal activities (Kossek et al., 2012).

Interruptions are known as stressors (Van Den Berg et al., 1996; Blank et al., 2020) and a stressor is followed by a stress reaction if the individual sees herself/himself not fit to handle the challenging situation in a way that it will end positively for them, typically manifesting itself in terms of high activated negative feelings, such as feeling strained (Ursin and Eriksen, 2010). This is critical because strain reactions have been described as pathogenic pathways leading to chronic physical and psychological impairments, especially in the long-term (Brosschot et al., 2005; Sonnentag and Frese, 2012).

Empirical studies show that interruptions relate to higher levels of strain and frustration (Mark et al., 2008), irritation (Baethge, 2013), sadness (Blank et al., 2020), and exhaustion (Pachler et al., 2018). In contrast to single interruptions, frequent interruptions are known to be more detrimental for workers’ wellbeing: If a person is interrupted over and over again, the stress reactions to every single interruption accumulate and lead to an accelerative increase of strain (Baethge et al., 2015). Consequently, a high frequency of interruptions is especially straining (Baethge et al., 2015), exhausting (Mark et al., 2008) and can potentially contribute to a reduced quality of life (Geurts and Sonnentag, 2006). Taken together, we thus add our first proposition as a complement to boundary theory (Ashforth et al., 2000).

*Proposition 1*: The higher the frequency and the lower the duration individuals engage in the work role during private time, the more likely they experience a strain effect and thus lower affective and psychosomatic wellbeing.

### 5.2. The gain effect of sustained work extending

Our review indicates that work extending’s duration does not relate to workers’ strain, exhaustion, and sleep quality. Moreover, and more surprisingly, our findings point towards a beneficial effect suggesting that a longer duration of work extending relates to better cognitive wellbeing. We propose this “gain effect” on the basis of a small number of studies that measured work extending via its duration. Workers’ cognitive wellbeing encompasses psychological enrichment and the exercise of problem-solving pondering. Research measuring work extending’s frequency did not show a relationship with these wellbeing indicators.

The gain of psychological wellbeing—or resources—due to engaging in work behaviours has been described as work-life-enrichment (Greenhaus and Powell, 2006). Thus, work-related experiences can enrich workers’ lives by enabling the acquisition of beneficial attitudes and skills (e.g., self-efficacy beliefs), which enriches them personally and is beneficial for their whole life, both the work and private life domains. This enrichment may be due to a learning mechanism: We argue that working outside of working hours and places enables workers to work without the frustrations, distractions, and time pressures of a typical work day. At home, in their private time, workers can decide autonomously how much time they want devote to a work task. Their engagement in a work activity for a certain length allows them to focus and supports a concentrated processing of the work task. Thus, in-depth processing of work-related information may lead to greater understanding and knowledge of a work task which are indicators for learning (Spreitzer et al., 2005).

Learning is an important indicator of thriving at work (Spreitzer et al., 2005), and empirical studies generally support a link between learning and wellbeing, especially for informal forms of learning (Jenkins, 2011). It refers to the perceptions that one is acquiring, as well as the ability to apply knowledge and skills (Elliott and Dweck, 1988). The adoption of skills while working contributes to character development and is useful to meet the challenges in private life (e.g., learning on the job how to look at a problem from different viewpoints which functions as a resource to accelerate the settlement of private conflicts) (Greenhaus and Powell, 2006; Hanson et al., 2006). Moreover, having a deep focus on a work task at home also means that the workers can ponder work problems, but without negative emotions accompanying these thoughts (Cropley and Zijlstra, 2011).

Consequently, work extending activities also potentially set the scene for learning which is beneficial for workers’ cognitive wellbeing. We do however acknowledge that it might backfire when longer work extending behaviours are carried out frequently and suggest that in order for learning processes to evolve, these behaviours need to be the exception rather than the norm. Thus, it would only be triggered by work extending behaviours of low frequency and high duration—leading to our second proposition as complement to boundary theory (Ashforth et al., 2000).

*Proposition 2*: The higher the duration and the lower the frequency individuals engage in the work role during private time, the more likely they experience a gain effect and thus higher cognitive wellbeing.

### 5.3. The loss-of-private-time effect of work extending

Time is a scarce resource and therefore, work extending behaviours inherently result in a loss of time for nonwork activities, such as recovery or family activities. Consequently, research showed that workers reported less psychological detachment and sleep quantity when they experienced higher frequency or higher duration of work extending. Although these relationships do not come as a surprise, they are important. Only in times when workers are not influenced by work in their actions and when rumination about work demands has stopped, can exhausted resources replenish (Sonnentag and Fritz, 2015). Moreover, our findings show that work extending’s frequency and duration relates to a conflict between work and private life. When workers engage in work outside their working hours and consequently fail to engage in private activities with family or friends or lack time to meet household duties, a conflict will arise between work and private life (Greenhaus and Beutell, 1985). Such a conflict is critical as it further relates to lower life satisfaction (Taşdelen-Karçkay and Bakalım, 2017), as well as to lower marital satisfaction of workers (Amstad et al., 2011) and their spouses (Bakker et al., 2009). Consequently, we state our third and last proposition as complement to boundary theory (Ashforth et al., 2000):

*Proposition 3*: The higher the duration and the higher the frequency individuals engage in the work role during private time, the more likely they experience a loss-of-private-time effect and thus lower social wellbeing as well as lower recovery from work.

### 5.4. Limitations

A number of limitations should also be noted. First, although we differentiated between between-person and within-person associations between work extending and wellbeing indicators, it remains unclear from the review results whether the associations between work extending and wellbeing were independent of other variables. More precisely, eleven articles reviewed did not provide information on the correlation between work extending and the wellbeing indicator but reported a regression analysis (e.g., Glavin et al., 2011 Bowen et al., 2018; Rau and Göllner, 2019) or reported the nature of the relationship only by text (Arlinghaus and Nachreiner, 2013; McCloskey, 2016). In the regression analysis, other predictors of wellbeing were considered simultaneaously (e.g., controlled for) with work extension. Most studies controlled for work characteristics such as job autonomy or workload (Schieman & Glavin, 2008 Glavin and Schieman, 2010; Schieman and Young, 2010 Wajcman et al., 2010; Glavin et al., 2011; Berkowsky, 2013; Bowen et al., 2018; Rau and Göllner, 2019), but also for technology-related demands and hassles (Barber and Jenkins, 2013), personal characteristics such as conscientiousness and job involvement (Barber and Jenkins, 2013), as well as sociodemographic variables (e.g., Glavin and Schieman, 2010; Schieman and Young, 2010 Wajcman et al., 2010; Glavin et al., 2011; Berkowsky, 2013). Consequently, we cannot be certain that the observed associations would have held if the effects of work extending had been isolated in these studies.

A second limitation concerns the information value of the empirical evidence due to a potential publication bias in favor of statistically significant findings. Due to guarantee a certain quality of the studies reviewed, we only considered manuscripts that underwent some kind of peer-review process, and most manuscripts were peer-reviewed in the course of publication in a journal. However, significant associations between variables may be more easily published than nonsignificant null findings, potentially inflating the ratio of positive/negative associations to null effects in our review. Nevertheless, it is notable that more than one quarter (i.e., 52) of the 189 accociations reviewed were null findings, which does not eliminate the danger of the publication bias but might at least mitigate it. Also, it is likely that the publication bias exists for both duration and frequency and thus, distinguishing between the two might limit its relevance.

Third, when it comes to our propositions, we remain cautious about causality because the vast majority of studies did not use a time-sensitive study design,. Due to theoretical considerations underlying the relationships, we opted for describing our findings as it seemed more likely to us than the other way around. Nonetheless, causality still needs to be tested in further studies.

### 5.5. Agenda for future research

To fully understand the contemporary phenomenon of technology-based work extending and its implications for workers’ wellbeing, more research is required. First, we are in need of empirical studies to specifically test our propositions. We mainly based our propositions on studies capturing either work extending’s frequency or duration, but argued that, in order to be able to predict its effect on wellbeing, it is not only important to know how many work extending episodes workers experience, but how long these episodes are. Consequently, future studies should capture information on both the frequency and the duration of workers’ behaviours in order to yield information on how long the work extending episodes lasted, or on how sustained the behaviours were (i.e., high duration and low frequency), respectively. Moreover, we advise future studies to apply longitudinal designs that allow for causality tests to be made in order to clarify the direction of our proposed effects.

Second, future studies should also include other possible temporal characteristics of technology-based work extending. Roe (2008), for example, describes multiple temporal features that define a behavioural phenomenon: its moment of onset (the starting point of the behaviour in time), its stability vs. instability (the behaviour stays the same or changes over time), its growth vs. decline (the behaviour gains intensity or loses intensity over time), and its recurrence vs. continuance. Drawing on stress theories, these features may have crucial effects on workers’ wellbeing: The first encounter of a stressor has a much stronger initial effect on workers than later encounters, because individuals need time to develop coping strategies, but also a longer exposure to a stressor can increase the impacts of the stressor on workers’ health (Frese and Zapf, 1988).

Third and finally, we are in need of studies that examine the time the potential effects of work extending on wellbeing indicators take to manifest themselves (Navarro et al., 2015). Although there have been some attempts in recent years to apply more time-sensitive study designs, especially diary studies, we still do not know much about the time between workers’ engagement in work extending behaviours and the change in their wellbeing. For example, although there is evidence that recurring work extending relates to increased levels of strain (Butts et al., 2015) and exhaustion (Schlachter, 2017) within 1 day, we do not know anything about their relationship within weeks and months of engaging in this behaviour. Moreover, we could not find any time-sensitive studies investigating the relationship between work extending and the wellbeing indicators psychological enrichment, psychosomatic health complaints, feeling recovered, perceived control over life, and (private) life satisfaction.

### 5.6. Practical implications

Being aware of the different effects of frequent vs. sustained work extending behaviours, organisations can strive to provide supportive conditions to ensure workers’ wellbeing. As the COVID-19 pandemic has accelerated the adoption of flexible work practices (Eurofound, 2021) and with it the blurring of the temporal and spatial boundaries between work and private life seems to have further increased. Thus, taking measures to support workers to maintain or adopt healthy behaviours outside working hours is especially important in this context.

First, as frequent work extending potentially strains workers and thus harms not only their wellbeing, but also their work engagement (Ferreira et al., 2019) and performance (Gillet et al., 2013), steps should be taken to minimize the frequency of work extending. This could be done, for example, by increasing clarity of organisational expectations. Organisations should ensure that workers neither feel implicit nor explicit pressure to extend work into their private lives by clarifying that workers are not expected to check their e-mails, take calls from co-workers or supervisors, or perform work tasks outside their working hours. Establishing clarity of organisational expectations on work extending not only changes workers’ behaviours, but also their general work satisfaction (Heißler, 2017). If organisations, however, do expect that their workers extend their work (sometimes), these occasions should be kept to a minimum and counted as regular working hours or on-call work.

Second, organisations should think about compensating workers for extra time. If the workers receive a temporal compensation for work extending, the loss-of-private-time effect of work extending may be buffered with workers facing a reduced risk of an impairment of their social wellbeing. If the workers receive monetary compensation for their work extending, it would be a morally correct approach (Eurofound and the International Labour Office, 2017). Moreover, an increased payment for increased effort helps to keep the balance between their efforts and rewards which has been described as crucial for wellbeing (Siegrist, 2002). Consequently, monetary rewards may have the potential to buffer the proposed strain effect of frequent work extending.

Third, our results reveal that sustained work extending potentially helps workers to gain cognitive resources and thus wellbeing, probably because this behaviour triggers a learning process. However, organisations should interpret this finding with great caution. In order to prevent a moral conflict and a legal gray area, it would be helpful for organisations to find a way to provide workers time within their working hours in which they can deal with a task in-depth for a certain period of time. Work (time) should be organized in a way that uninterrupted work activities could also be experienced within the realms of paid working time. This way, workers might profit from their deep focus on a work task and gain cognitive resources helping them to flourish, without having to sacrifice private time which they also need to recover from their regular work efforts.

## 6. Conclusion

Considering the growing prevalence of technology-based work extending, workers as well as organisations need to be aware of the psychological implications this behaviour has, especially when it comes to consequences for wellbeing. Our systematic literature review on the relationship between technology-based work extending and workers’ wellbeing indicates that work extending always causes a loss of private time and thus potentially reduces recovery and social wellbeing due to conflicts between work and private life. However, by grasping work extending a contemporary behavioural phenomenon that can have different (and varying) temporal characteristics, we were able to derive two more specific propositions from our findings: First, we propose a strain effect following a higher frequency of work extending. Shorter but frequent contacts with work outside working hours cause potential interruptions during the enactment of a private life role, and frequent interruptions increase workers’ negative affect such as feelings of strain and exhaustion. Second, we propose a gain effect of sustained work extending. Longer, less frequent contacts with work can be used to deepen focus on work for learning and growth, which has a positive impact on workers’ cognitive wellbeing. However, more empirical research is necessary to further test these propositions.

## Author contributions

JS and MH-T had the idea and designed the study and wrote the manuscript. JS collected and analyzed the data. CK provided feedback throughout the process. All authors contributed to the article and approved the submitted version.

## Conflict of interest

The authors declare that the research was conducted in the absence of any commercial or financial relationships that could be construed as a potential conflict of interest.

## Publisher’s note

All claims expressed in this article are solely those of the authors and do not necessarily represent those of their affiliated organizations, or those of the publisher, the editors and the reviewers. Any product that may be evaluated in this article, or claim that may be made by its manufacturer, is not guaranteed or endorsed by the publisher.
